# A Magnetoelectric Distance Estimation System for Relative Human Motion Tracking

**DOI:** 10.3390/s25020495

**Published:** 2025-01-16

**Authors:** Johannes Hoffmann, Henrik Wolframm, Erik Engelhardt, Moritz Boueke, Tobias Schmidt, Julius Welzel, Michael Höft, Walter Maetzler, Gerhard Schmidt

**Affiliations:** 1 Department of Electrical and Information Engineering, Kiel University, 24143 Kiel, Germany; jph@tf.uni-kiel.de (J.H.); hewo@tf.uni-kiel.de (H.W.); eren@tf.uni-kiel.de (E.E.); mobo@tf.uni-kiel.de (M.B.); tsc@tf.uni-kiel.de (T.S.); mh@tf.uni-kiel.de (M.H.); 2 Department of Neurology, Kiel University, 24105 Kiel, Germany; j.welzel@neurologie.uni-kiel.de (J.W.); w.maetzler@neurologie.uni-kiel.de (W.M.)

**Keywords:** digital signal processing, gait analysis, magnetic motion tracking, magnetoelectric sensor, technical validation

## Abstract

Clinical motion analysis plays an important role in the diagnosis and treatment of mobility-limiting diseases. Within this assessment, relative (point-to-point) tracking of extremities could benefit from increased accuracy. Given the limitations of current wearable sensor technology, supplementary spatial data such as distance estimates could provide added value. Therefore, we propose a distributed magnetic tracking system based on early-stage demonstrators of novel magnetoelectric (ME) sensors. The system consists of two body-worn magnetic actuators and four ME sensor arrays (body-worn and fixed). It is enabled by a comprehensive signal processing framework with sensor-specific signal enhancement and a gradient descent-based system calibration. As a pilot study, we evaluated the technical feasibility of the described system for motion tracking in general (Scenario A) and for operation during treadmill walking (Scenario B). At distances of up to 60 cm, we achieved a mean absolute distance error of 0.4 cm during gait experiments. Our results show that the modular system is capable of centimeter-level motion tracking of the lower extremities during treadmill walking and should therefore be investigated for clinical gait parameter assessment.

## 1. Introduction

Mobility-limiting diseases such as Parkinson’s disease (PD) have a significant impact on patients’ daily activities. Postural control is impaired even in the early stages of PD, leading to a more cautious gait (reduction of forward progression) and in later stages to instability and increased risk of falls [1]. Diagnosis and assessment of treatment success rely on standardized clinical routines and test scales as well as quantitative biomechanical measures [2]. This includes spatiotemporal gait features such as stride lengths and times, kinematic features such as angles, and kinetic features such as joint moments and forces [3].

A comprehensive movement assessment requires consideration of capacity (’How good can you perform?’) and performance aspects (’How good do you usually perform?’) [4]. Capacity can be examined by having the patient do supervised exercises such as walking tasks [5]. Assessment with sensor systems such as optical motion capture (OMC) or multi-device setups of inertial measurement units (IMUs) is applicable in this scenario. Performance measures focus on unsupervised movements, e.g., in the usual environment of the patient, and may differ significantly from capacity measures. Home assessment setups are typically more minimal in nature, e.g., consisting of few body-worn IMUs [6]. The assessment of gait stability by step length or width is possible to some extent with these setups, but accuracy is limited compared to the OMC gold standard [7,8,9].

A wide range of supplementary sensors have been proposed that can directly provide position or distance data to overcome these limitations. Light Detection and Ranging (LiDAR) [10] or millimeter wave radar [11] methods provide absolute position data as a more portable or affordable alternative to OMC but are also stationary. Time-of-arrival methods by ultra-wideband (UWB) transmission have also been proposed for centimeter-accurate position tracking, but also have some limitations due to multipath propagation and often require fixed anchor points [12]. Magnetic methods in human motion tracking are also common but usually limited to the geomagnetometers of IMUs (passive) [13]. Nevertheless, there have been some efforts to use active (artificial) fields for device localization [14], wearable applications [15], or indoor navigation [16] in both pilot studies and commercial products [17]. Often, non-linear optimization approaches are used as they are more flexible with respect to actuator–sensor configuration at the cost of computational complexity [18]. Analytical solutions for point-to-point tracking require specific orthogonal configurations but are straightforward to compute, making them suitable for embedded hardware such as in wearable devices [19,20]. Magnetic dipole fields decay with the third power of distance, which limits the range depending on actuator power and sensor sensitivity, but signals are not affected by multipath propagation and the Doppler effect [21]. Non-line-of-sight (NLOS) tracking is possible because non-metallic objects (such as the human body) are transparent to magnetic fields, preventing occlusion compared to OMC systems. However, hard (or soft) magnetic objects can interfere with the measurements, but this error can be corrected in some scenarios [22]. Operating at higher frequencies ensures separation of signals from stray fields such as the geomagnetic field. Any active magnetic motion tracking approach requires a magnetic field source, subsequently referred to as a magnetic actuator. While coils are most commonly used, there are other options such as oscillating permanent magnet cantilevers [23]. With a division of magnetic signals by time [15], frequency [20], or code [16], networks of many devices are feasible, in which each sensor node tracks its pose relative to each actuator node. Distributed tracking has potential advantages over a centralized magnetic actuator because the distances are smaller and therefore less affected by field decay and magnetic objects. Magnetoelectric (ME) sensors are a relatively new type of magnetometer based on the magnetoelectric effect [24,25]. A magnetostrictive layer exposed to a magnetic field contracts, causing the attached piezoelectric layer to generate a corresponding electrical signal. ME sensors have not yet found their way into broader applications, while fundamental limits [26], sensor design [27,28], and applications in the biomedical field [29,30] are being actively researched. The sensor principle applied in this study uses 3 × 1 mm cantilevers to form a resonant magnetometer sensitive to AC magnetic fields near their resonance frequency of about 7.7 kHz. The underlying exchange biased magnetoelectric composites are described in [31] while the current sensor fabrication process is closely related to [32]. With a sensitivity in the range of 10 pT/$\sqrt{\mathrm{Hz}}$ at resonance, the early-stage ME sensor is comparable to commercial fluxgate sensors [33] or magnetoimpedance (MI) sensors [34], which are other candidates for the described application. With further downscaling, a mature version of these sensor elements could be directly integrated into micro-electromechanical systems (MEMS) due to the similar fabrication process, which is very attractive for sensor fusion applications, e.g., with IMUs.

While we have previously demonstrated the application of ME sensors in both 1D (x-position only) [35] and full 6D (position and orientation) ([36] updated) motion tracking, this study focuses exclusively on the estimation of distances (vector norm of relative position). Since the sensor type is not yet well established (early-stage demonstrators), operating a multitude of ME sensors in an application environment presents a significant challenge. Additionally, the sensors are to some extent sensitive to acoustic and mechanical noise [37], which has implications for body-worn sensors. With this in mind, we investigate magnetic motion tracking not only for arbitrary movements (Scenario A), but also in a scaled-up system of two actuators and four sensor arrays for walking on a treadmill (Scenario B) at velocities of 0.5 m/s and 1 m/s. In that, the placement of actuator and sensor nodes on the lower extremities is chosen for technical reasons, so that the system has to cope with the distances, bandwidths, and potential disturbances from walking while also ensuring coverage by an OMC reference system. The obtained motion data are publicly accessible (see Data Availability Statement).

In this study, we describe the novel ME sensor-based motion tracking system in detail and investigate its distance estimation performance under arbitrary motion conditions and walking conditions. The main goal is to validate the system’s viability for further experiments focused on the assessment of specific clinical motor markers. However, such experiments are beyond the scope of this study as they would require dedicated detection and analysis algorithms.

## 2. Materials and Methods

**Figure 1:**
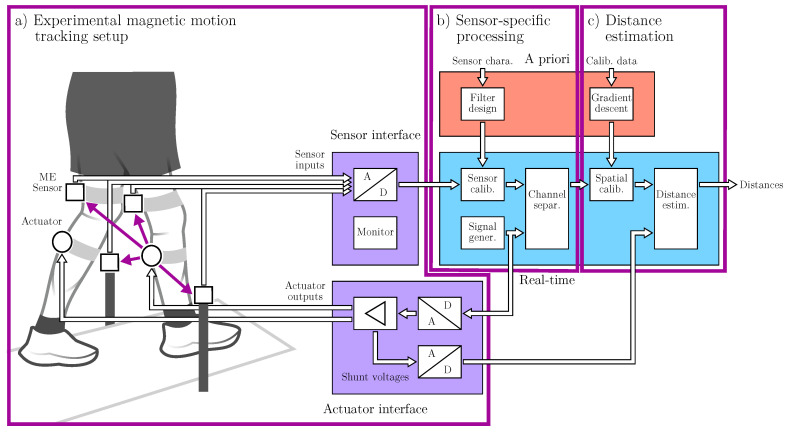
Overall signal processing pipeline of the magnetic motion tracking system. This includes the experimental hardware setup (**a**), the sensor-specific processing and enhancement (**b**), and the distance estimation and spatial calibration (**c**).

This section is divided into three subsections covering specific parts of the overall magnetic motion tracking system. The ’experimental motion tracking setup’ part (Figure 1a) focuses on the preliminary considerations regarding the gait analysis scenario as well as the specifically designed sensing hardware required for it. The ’sensor-specific signal processing and enhancement’ part (Figure 1b) describes the low-level measurement and signal processing aspects necessary to drive the actuators and obtain high-quality sensor readings. Finally, the ’distance estimation and spatial calibration’ part (Figure 1c) covers the digital signal processing aspects of the estimation itself as well as additions toward a comprehensive calibration algorithm.

### 2.1. Experimental Motion Tracking Setup

#### 2.1.1. Overview

**Figure 2:**
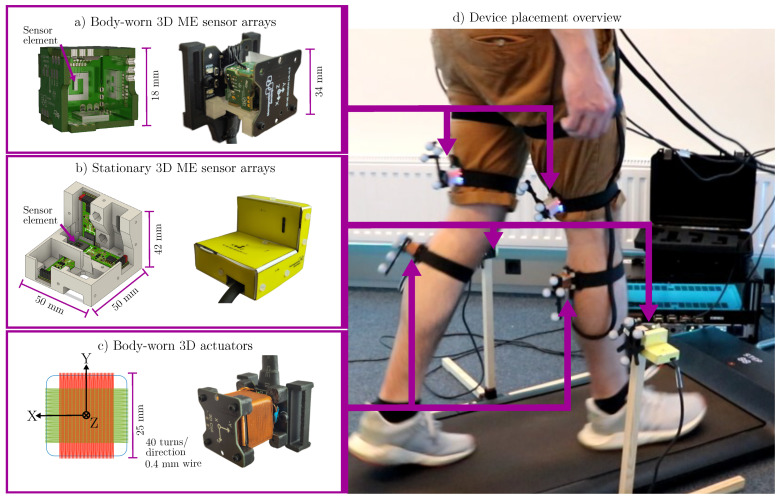
Magnetic motion tracking setup for gait experiments. Two body-worn sensor nodes (**a**) were attached to the subject’s thighs. Two stationary sensor nodes (**b**) were mounted left and right of the treadmill. Two actuator nodes (**c**) were attached to the subject’s shanks. The whole experimental setup is depicted in (**d**).

The overall setup (Figure 2) consisted of the magnetic and the optical tracking system as well as supplementary hardware. The treadmill (Kingston Walking Pad A1) was placed inside of a semicircular six-camera optical motion capture volume (Qualisys Miqus M3) with rigid bodies made of 19 mm retroreflective markers attached to all tracked devices. A priori knowledge of the sensors’ and actuators’ relative position toward the markers was used to compute a reference sensor trajectory as ground truth.

Figure 1 serves as a block diagram to visualize the applied intra-component signal flow and processing scheme, which is centered around the magnetic sensors and actuators. The actuators were driven by sinusoidal signals near the ME sensors’ resonance to generate corresponding magnetic AC fields. To separate the individual actuators at the sensor level, a frequency-division multiple access (OFDMA) scheme was used in the frequency range from 7425 Hz to 7675 Hz (50 Hz spacing). We chose a bandwidth of 25 Hz to cover both the fundamental frequency components during walking as well as the corresponding harmonics with some margin. This was implemented with matched filters of 40 ms length. The filters were applied once per frame at 48 kHz input sample rate and 512 samples per frame, which resulted in an output sample rate of 93.75 Hz. The software part (cf. Figure 1b,c) of the system was implemented in the Kiel Real-time Application Toolkit [38] to enable live feedback and monitoring during experiments with audio interfaces (RME Fireface UFX+ and RME Fireface 802) acting as AD/DA converters.

#### 2.1.2. Cube Sensor Arrays

The cube sensor array (see Figure 2a) was comprised of three identical sensor systems, which were combined into a cube-shaped configuration. The objective was to integrate three orthogonally aligned sensitive elements in the most compact manner possible. Each individual sensor system was comprised of two units: a printed circuit board (PCB) for mounting the sensitive element and another PCB for the charge amplifier. The geometry of each PCB was identical and selected to enable solder connections at right angles between them (castellated holes to pads) at their edges. The resulting array contained the complete amplifier circuits and the sensitive elements inside the cube, where they are protected from accidental contact and electrically shielded by a common ground plane. The external circuitry, signal lines, and common power supply of the system were connected via connectors on the outside of the cube.

#### 2.1.3. L-Shaped Sensor Arrays

The L-shaped sensor array (Figure 2b) was conceptualized as an easy-to-implement arrangement of existing ME sensors in a robust triaxial configuration. Three sensor PCBs were placed inside a 3D-printed body. Similar to the cube design, an outer shell made of PCB material was wrapped around the body for electric shielding purposes. With a significantly larger form factor compared to the cube sensor design, these devices are less suitable for body-worn applications, but can still be used as stationary reference sensors. In this study, two of these sensor arrays were mounted to the left and right of the treadmill (Figure 2d) on 50 cm high stands made of wood and ABS plastic.

#### 2.1.4. Actuators

The analytical estimation algorithm applied in this approach also required triaxial magnetic actuators (actuator nodes), which were created by winding three separate square coils on a common 3D-printed body (Figure 2c). Each actuator node was connected to the amplifiers (composite configuration [35]) in the actuator interface by coaxial cables to drive the channels individually. In the study, two actuator nodes (i.e., six individual channels) were driven with sinusoidal signals of 1.2 V and approximately 480 mA (both RMS), with slight variations for each channel due to manufacturing tolerances. The current was monitored in real time during measurements with a 100 mΩ shunt integrated with the amplifiers. The magnetic flux densities measured (depending on the distance) were in the nT to μT range.

### 2.2. Sensor-Specific Signal Processing and Enhancement

The applied ME cantilever sensors show a resonant behavior (first bending mode, 7.4 kHz to 7.8 kHz). Consequently, an inverse correction filter (equalizer) was required to obtain accurate magnetic field measurements over a broader frequency range. While each sensor was characterized after fabrication in a state-of-the-art characterization setup including magnetic shielding and acoustic dampening [25], these measurements were targeted at obtaining general-purpose sensor metrics, insufficient for accurate motion tracking applications. Therefore, we propose an adapted calibration scheme.

In general, the amplitude response |H| of a linear and time-invariant (LTI) system is linked to the quotient of output power spectral density $S_{\mathrm{yy}}$ and input power spectral density $S_{\mathrm{vv}}$ [39]. For the amplitude spectral densities, the following applies:

(1) $$|H\left(e^{j\Omega }\right)|=\frac{\sqrt{S_{\mathrm{yy}}\left(\Omega \right)}}{\sqrt{S_{\mathrm{vv}}\left(\Omega \right)}}=\frac{A_{\mathrm{yy}}\left(\Omega \right)}{A_{\mathrm{vv}}\left(\Omega \right)}.$$

**Figure 3:**
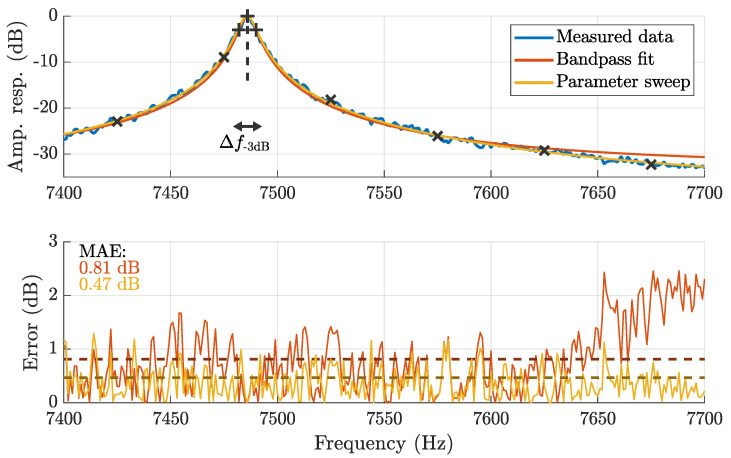
Exemplary comparison of the parameter sweep filter design method on ME sensor characterization data compared to standard bandpass fitting with mean absolute error (MAE) for both cases. The relevant points for the bandpass fit are denoted with ‘+’ and the actuator frequencies are denoted with ‘×’.

For characterization purposes, the sensor was excited with band-limited noise [40] in the frequency range of 6 kHz to 9 kHz inside of a Helmholtz coil. By dividing the voltage spectrum measured with the ME sensor by the locally linearized magnetic spectrum measured with a previously characterized reference sensor (Aichi Steel MI-CB-1DJ [34]), the ME sensor’s sensitivity over frequency is available. This can be expressed as a normalized amplitude response (cf., Figure 3) and a peak sensitivity value in volts per tesla. Based on the resulting spectrum, parametric bandpass characteristics, such as center frequency and −3 dB bandwidth, are available (cf., Figure 3). IIR peak (boost) filters were previously identified as a suitable fit for this type of ME sensor [35]. Such a parametric filter design requires center frequency, bandwidth, and an optional gain parameter that sets an appropriate attenuation limit for frequencies far away from the center frequency [41].

ME sensors have been previously used for motion sensing experiments but usually with only a few excitation signals, so that a rough equalization based on the bandpass fit and some manual optimization was considered sufficient (Figure 3). However, as this study uses twelve sensors and six simultaneous excitation signals with 50 Hz spacing between each pair, a more accurate and automated approach was desired. Consequently, the bandpass fit parameters and the estimated gain ($f_{0}$, ∆$f_{0}$, and $G_{0}$) were used as initial values for a parameter sweep optimization scheme that considers the relevant spectrum from 7.4 kHz to 7.7 kHz instead of only three dedicated frequency points. The sweep involved all combinations of center frequencies in the range $f_{0}\;\pm \;$ 3 Hz (0.1 Hz resolution), bandwidths in the range $∆f_{0}\;\pm \;$ 6 Hz (0.1 Hz resolution), and gains in the range of $G_{0}$ −15 dB to $G_{0}$ (1 dB resolution). For each parameter combination *i*, the amplitude response ${\hat{H}}_{i}$ was computed and compared to the measured noise spectrum to find the combination with the least mean absolute error (MAE):

(2) $$\sum_{\mu }||H(e^{j\Omega_{\mu }})|-|{\hat{H}}_{i}(e^{j\Omega_{\mu }})||\to \min.$$

This computation was applied based on the dB values to achieve a logarithmic weighting. Figure 3 illustrates the improved fit for an exemplary sensor (Sensor node 0 - Channel x) as visible from both the amplitude and the error spectrum. Consequently, this method was applied to design equalizing filters for all involved ME sensors. More details are available in Table A1 of Appendix A.

### 2.3. Distance Estimation and Spatial Calibration

In general, we consider the distance *r* as the vector norm of the relative position between the actuator and sensor node with position vectors ${\vec{r}}_{a}$ and ${\vec{r}}_{s}$:

(3) $$r=|{\vec{r}}_{s}\;-\;{\vec{r}}_{a}|.$$

**Figure 4:**
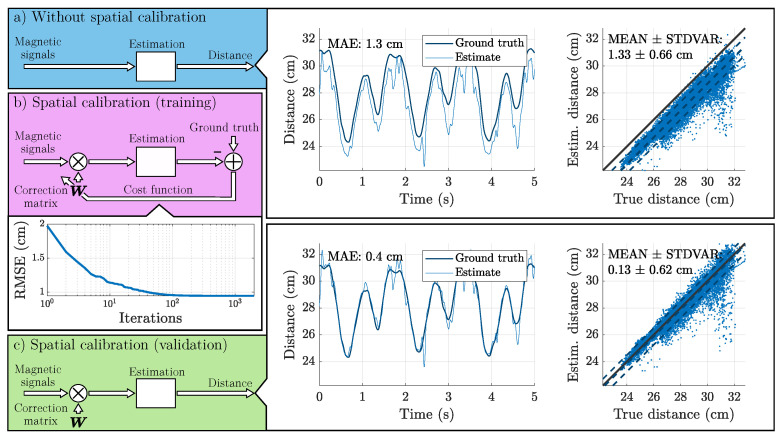
Fundamental mechanics of the spatial calibration process explained on results from Scenario B. Standard estimation pipeline without spatial calibration (**a**). Multiplication with a correction matrix and optimization of this matrix on separate data (training) in a gradient descent approach to minimize the deviation from the ground truth data (**b**). Use of the pre-trained correction matrix for validation (**c**). The central dashed line in the plots of estimated distance versus true distance represents the mean error by which the estimate deviates from an ideal estimator (solid line). The additional lines above and below represent the mean error plus and minus one standard deviation.

As illustrated in Figure 4a for a single actuator–sensor pair, the estimation algorithm computes a distance estimate based on the measured magnetic signals. While the fundamental estimation algorithm has been published previously [36], we briefly reiterate the parts that are crucial for the novel calibration process. In general, a 3 × 3 magnetic field matrix is given for each actuator–sensor pair. For an ideal point sensor array, the normalized magnetic field matrix Θ depends on the sensor node’s position vector $\vec{r}$ and orientation matrix R. It is equal to a product of the decay $\frac{1}{r^{3}}$, the orientation matrix, and a directivity matrix Φ. Both Θ and Φ can be decomposed into three column vectors ${\vec{\theta }}_{i}$ or ${\vec{\phi }}_{i}$. Here, ${\vec{\theta }}_{i}$ corresponds to the magnetic vector field generated by one of the individual coils in an actuator node:

(4) $$\underset{\left[\begin{array}{c} {\vec{\theta }}_{x},\;{\vec{\theta }}_{y},\;{\vec{\theta }}_{z} \end{array}\right]}{\underset{︸}{\Theta (\vec{r},R)}}\;\;\;\;\;=\frac{1}{r^{3}}\;\;\;\cdot \;\;\;R\;\;\;\cdot \;\;\;\;\;\;\underset{\left[\begin{array}{c} {\vec{\phi }}_{x},\;{\vec{\phi }}_{y},\;{\vec{\phi }}_{z} \end{array}\right]}{\underset{︸}{\Phi ({\vec{e}}_{r})}}\;.$$

Furthermore, each of the column vectors ${\vec{\theta }}_{i}$ represents three scalars, which are available as the x, y, and z components of this coil’s magnetic vector field at the sensor. The matrix Φ represents the position dependency of the vector field with the normalized position vector $|{\vec{e}}_{r}|$ = 1:

(5) $${\vec{\theta }}_{x}=\left[\begin{array}{c} \theta_{\mathrm{xx}}\\ \theta_{\mathrm{yx}}\\ \theta_{\mathrm{zx}} \end{array}\right],\;\Phi \left({\vec{e}}_{r}\right)=\frac{1}{2}\left[\begin{array}{ccc} 3x^{2}-1 & 3xy & 3xz\\ 3xy & 3y^{2}-1 & 3yz\\ 3xz & 3yz & 3z^{2}-1 \end{array}\right].$$

In the following steps, we solve for the distance ($r=|\vec{r}|$) by exploiting properties of the Frobenius norm [42], which has a low computational complexity and is differentiable:

(6) $${∥\Theta (\vec{r},R)∥}_{F}=\frac{1}{r^{3}}\;\;{∥R\;\Phi ({\vec{e}}_{r})∥}_{F}\Rightarrow r=\sqrt[{3}]{\frac{∥R\;\Phi \left({\vec{e}}_{r}\right){∥}_{F}}{∥\Theta \left(\vec{r},\;R\right){∥}_{F}}}.$$

The rotation matrix can be disregarded since it does not affect the norm by definition. Additionally, the norm of Φ is a known constant, cf., structure in (5). So, only the sixth root of a constant over a square sum remains:

(7) $$\begin{array}{cc} r & =\sqrt[{3}]{\frac{∥\Phi \left({\vec{e}}_{r}\right){∥}_{F}}{∥\Theta \left(\vec{r},\;R\right){∥}_{F}}}=\frac{\sqrt[{6}]{3/2}}{\sqrt[{3}]{∥\Theta \left(\vec{r},\;R\right){∥}_{F}}} \end{array}$$

(8) $$\begin{array}{cc} \; & =\frac{\sqrt[{6}]{3/2}}{\sqrt[{6}]{\theta_{\mathrm{xx}}^{2}+\theta_{\mathrm{xy}}^{2}+\theta_{\mathrm{xz}}^{2}+\ldots }}. \end{array}$$

The resulting distance estimate can be subtracted from the ground truth distance (optical reference) to obtain error metrics. Figure 4a also includes exemplary results for this scenario as a time signal and a distance estimate over the ground truth distance plot. From these signals, a systematic error between the estimate and the ground truth is clearly visible. The main reason is assumed to be a deviation between the sensor’s long and sensitive axis [43], which is slightly different for each sensor (not ideally orthogonal array). Additional inaccuracies can be attributed to sensor deviations from the ideal bandpass behavior and fabrication tolerances, such as misalignment of the sensor elements during array assembly and manufacturing of the coils (hand-made). This corresponds to a distortion of the magnetic field matrix of the real sensor $\tilde{\Theta }$ compared to the matrix of the ideal sensor Θ. Therefore, we introduce a weighting matrix W that represents these distortions:

(9) $$W\tilde{\Theta }\left(\vec{r},R\right)=\Theta \left(\vec{r},R\right)=\frac{1}{r^{3}}\;R\;\Phi \left({\vec{e}}_{r}\right).$$

However, each field vector ${\tilde{\vec{\theta }}}_{i}$ is potentially distorted in a different way, e.g., due to frequency-dependent behavior not fully covered by the equalizer. Consequently, we further generalize our approach by using not one single weighting matrix but individual weighting matrices for each magnetic field vector, e.g., $W_{x}$ for ${\tilde{\vec{\theta }}}_{x}$ instead of W:

(10) $$W_{x}{\tilde{\vec{\theta }}}_{x}\left(\vec{r},R\right)=\frac{1}{r^{3}}\;R\;{\vec{\phi }}_{x}\left({\vec{e}}_{r}\right).$$

The distance *r* can now be derived similarly to (6) and (7):

(11) $$\begin{array}{cc} r & =\sqrt[{3}]{\frac{∥R\;\Phi \left({\vec{e}}_{r}\right){∥}_{F}}{∥\left[W_{x}{\tilde{\vec{\theta }}}_{x},\;W_{y}{\tilde{\vec{\theta }}}_{y},\;W_{z}{\tilde{\vec{\theta }}}_{z}\right]{∥}_{F}}} \end{array}$$

(12) $$\begin{array}{cc} \; & =\frac{\sqrt[{6}]{3/2}}{\sqrt[{6}]{{\left(w_{\mathrm{xx}}^{x}{\tilde{\theta }}_{\mathrm{xx}}+w_{\mathrm{xy}}^{x}{\tilde{\theta }}_{\mathrm{yx}}+w_{\mathrm{xz}}^{x}{\tilde{\theta }}_{\mathrm{zx}}\right)}^{2}+\ldots }}. \end{array}$$

At this stage, we leave the detailed notation focused on magnetic fields for a more abstract optimization approach notation. Consequently, we reshape the three weighting matrices for one actuator–sensor combination into a single weight vector with 3·9=27 elements:

(13) $$w=\left[\begin{array}{c} w_{0}\\ \vdots \\ w_{26} \end{array}\right]={\left[\begin{array}{c} w_{\mathrm{xx}}^{x},w_{\mathrm{xy}}^{x},\cdots ,w_{\mathrm{xx}}^{y},\cdots ,w_{\mathrm{zz}}^{z} \end{array}\right]}^{⊺}.$$

The ideal w would perfectly reconstruct the magnetic field matrix $\tilde{\Theta }$ according to the model, but it is unknown. Instead, we introduce an iterative optimization process to estimate $\hat{w}$, in which the cost function *c* is the mean squared error (MSE) of the distance estimate $\hat{r}$ and the ground truth distance (optical reference) *r* at iteration *k*. Both signals are time-dependent with index *n* and signal length *N* as we assume a movement scenario:

(14) $$c^{\left(k\right)}=\frac{1}{N}\sum_{n=0}^{N-1}{\left(r\left(n\right)-\hat{r}\left(n,{\hat{w}}^{\left(k\right)}\right)\right)}^{2}\to \min.$$

An iterative adaptation of $\hat{w}$ can now be computed based on a gradient descent approach [44] by differentiating the cost function by the individual weights with step size α:

(15) $${\hat{w}}^{\left(k+1\right)}={\hat{w}}^{\left(k\right)}-\alpha \left[\frac{\partial c^{\left(k\right)}}{\partial {\hat{w}}^{\left(k\right)}}\right].$$

Equations (14) and (15) form the outer optimization loop that is highlighted in Figure 4b. The square root of the cost function (RMSE) decreases with each iteration. The process terminates once the resulting improvement is less than 1 × 10^−15^ m^2^ or 2000 iterations have passed.

Backtracking line search is a commonly used method to ensure an appropriate step size during an optimization process [45]. It acts as an inner optimization loop during each iteration *k*, by setting the step size $\alpha^{\left(m\right)}$ to an initial value and then subsequently decreasing it by the parameter ρ = 0.8:

(16) $$\alpha^{\left(0\right)}=1000,\;\alpha^{\left(m+1\right)}:=\rho \;\alpha^{\left(m\right)}.$$

This iterative process terminates once the Armijo sufficient decrease condition is fulfilled (with σ = $1\;\times \;10^{-4}$):

(17) $$\underset{\mathrm{with}\;\alpha^{\left(m\right)}}{\underset{︸}{c^{\left(k+1\right)}}}\;\leq c^{\left(k\right)}-\sigma \;\alpha^{\left(m\right)}{\left[\frac{\partial c^{\left(k\right)}}{\partial {\hat{w}}^{\left(k\right)}}\right]}^{⊺}\;\;\cdot \;\left[\frac{\partial c^{\left(k\right)}}{\partial {\hat{w}}^{\left(k\right)}}\right].$$

Finally, the estimated correction vector $\hat{w}$ can be applied on a different dataset (validation, Figure 4c) to verify that a reduction in error is achieved. In the example, the MAE has decreased from 1.33 cm to 0.13 cm, which is mainly the result of a decrease in the error signal’s mean value.

## 3. Results

### 3.1. Acquisition of Datasets

A set of measurements was conducted to evaluate the system’s performance in two different scenarios against a gold-standard optical motion capture system. Separate datasets were recorded for training (optimization of the spatial calibration) and validation.

Scenario A

This scenario is purely technical in nature and does not involve human subjects. Similar to the wand-based calibration of an optical motion capture system, the actuator was moved arbitrarily around the sensor. This procedure was carried out twice for each actuator–sensor combination (80 s to 160 s for training and 60 s for validation).

Scenario B

This scenario involved a technical demonstration of the system under conditions similar to routines performed in clinical gait analyses. Five healthy subjects (male, age: 25 to 31) walked on a treadmill at speeds of 0.5 m/s and 1 m/s while being equipped with the full setup of magnetic actuators and sensor arrays. We conducted two recordings per subject with a duration of 120 s per speed. Data from two subjects were used exclusively for training (calibration), and data from three subjects were used exclusively for validation. The latter are referred to as Subjects 1, 2, and 3 in the exemplary signals section.

### 3.2. Qualitative Description of Exemplary Signals

**Figure 5:**
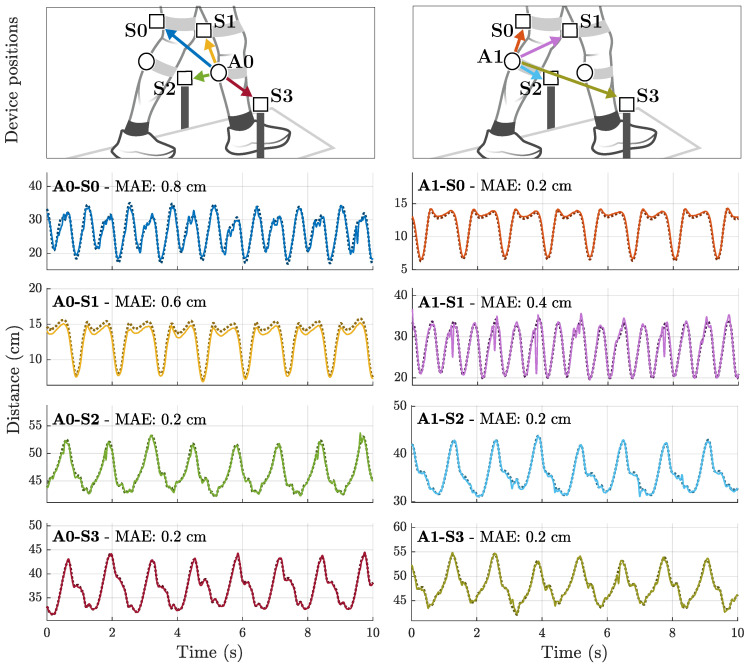
Exemplary signals of gait experiments (Scenario B) with the full magnetic motion tracking system. Optical reference signals (ground truth) in darker color and dotted.

Figure 5 displays how the distance signals during gait (Scenario B) look like in principle, to give the reader a better understanding of what to expect. The plot contains the first section of the recorded time signals for Subject 3 during treadmill walking at 0.5 m/s.

The upper left signal (A0-S0) represents the distance between the right shank and the left thigh. The signal is roughly sinusoidal with a slight deviation in amplitude between two successive half-waves. Since both limbs move individually, the fundamental frequency is twice as high compared to the absolute movement as in A0-S2. The signal A0-S1 represents the movement between right shank and thigh. Extension and flexion of the knee are clearly visible. The lower left signals show the absolute distance of the right shank towards the stationary sensors. Both signals show qualitative similarity with reduced amplitude in A0-S3 because this sensor node is closer to the left shank. The signals of the left actuator node (A1) are clearly symmetrical to the right actuator node (A0).

**Figure 6:**
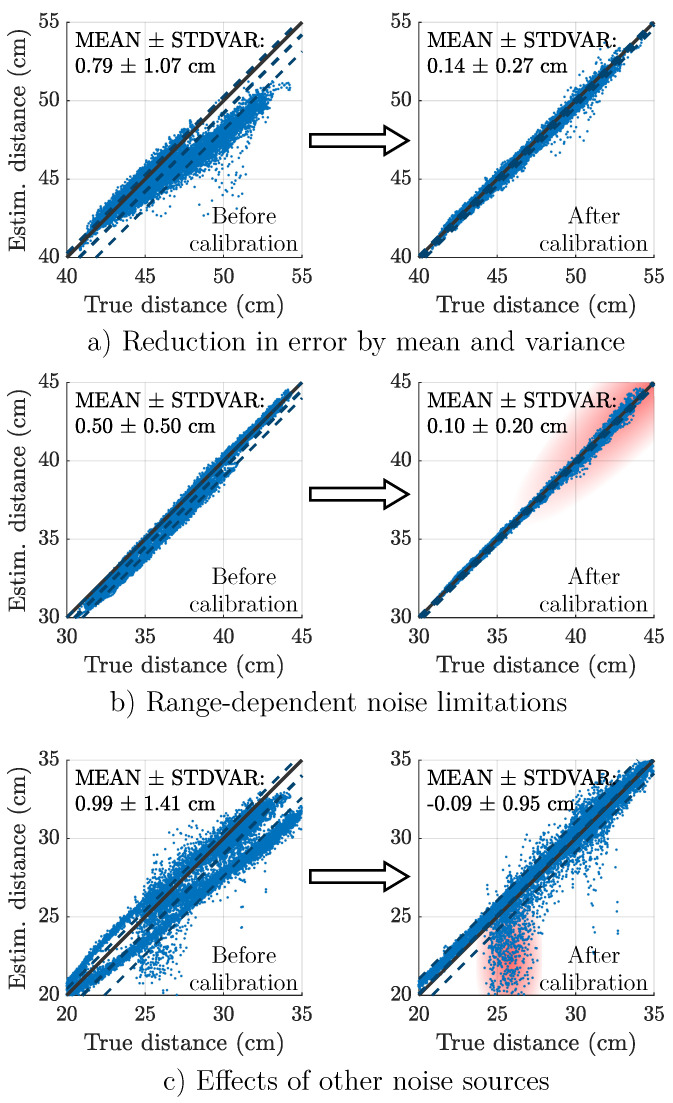
Selected results to highlight capabilities and limitations of the spatial calibration approach during gait (Scenario B). The central dashed line represents the mean error by which the estimate deviates from an ideal estimator (solid line). The additional lines above and below represent the mean error plus and minus one standard deviation. Systematic correction of the error signal in mean and variance (**a**). Limited ability to reduce error at higher distances as highlighted in red (**b**). Limited ability to reduce error caused by other noise sources as highlighted in red (**c**).

Figure 6 displays additional selected results from Scenario B as distance-over-distance plots. The results are given before and after spatial calibration for a detailed comparison of visuals and error metrics. Figure 6a shows the results for the actuator–sensor pair A0-S2 of Subject 2 during 1 m/s walking. Figure 6b shows the results for the actuator–sensor pair A0-S3 of Subject 2 during 1 m/s walking. Figure 6c shows the results for the actuator–sensor pair A0-S0 of Subject 2 during 0.5 m/s walking.

### 3.3. Overview on System Performance

Table 1 summarizes the obtained errors to give an overview of the available performance and the effectiveness of the spatial calibration mechanism. Each row represents one of the training conditions (without calibration, training on Scenario A data only, training on Scenario B data only, training on data from both scenarios). The columns (Scenario A and Scenario B) represent the data that were used for validation. The mean (averaged) MAE over all trials and all actuator–sensor pairs in the validation data was chosen as the main performance metric. In addition, the MAE of the worst-performing actuator–sensor pair (max. MAE) in any of the included experiments is shown to highlight the upper performance limit. While training only on the Scenario A data results in a slight reduction of the mean and maximum MAE on the Scenario A dataset, it does not result in improvements on the Scenario B dataset. Conversely, Scenario B training already achieves high performance on the Scenario B dataset, but even a slight performance decrease on both metrics of the Scenario A dataset. Training with data from both scenarios achieves the best recorded performance on the scenario A dataset and even a slight improvement on the worst-performing actuator–sensor pair of the Scenario B dataset compared to Scenario A training alone.

## 4. Discussion

In this paper, we evaluated the distance estimation performance of a ME sensor-based magnetic motion tracking system in two different scenarios. Table 1 summarizes the results for the arbitrary movements in Scenario A and the gait experiments in Scenario B. For the uncalibrated case, we achieved results of 1.4 cm and 1.0 cm MAE, which generally confirm the applied ME sensor model. The spatial calibration method leads to further accuracy improvements in both scenarios. Figure 6 provides selected results for a closer look at specific capabilities and limitations of the method.

The calibration explainer plot Figure 4 highlights a performance increase based on a reduction of the mean error, while the standard deviation of the error remains almost the same. In contrast, Figure 6a emphasizes the ability to reshape the estimated distance versus true distance plot with a significant reduction in mean and standard deviation. For both signals, we assume that systematic model errors (e.g., an inaccurate weighting of the sensor signals) were decreased, which also led to a decrease in MAE.

While Figure 6b also exhibits an error reduction at lower distances, it shows an increase near 45 cm. Since accuracy generally decreases with distance (due to signal-to-noise ratio), the effective tracking range for each actuator–sensor pair varies within a corridor of 40 cm to 60 cm. The spatial calibration can not correct this systematic increase in noise. The reduced performance gain by spatial calibration in Scenario A (larger distances compared to Scenario B) underlines this limitation of range.

In a third example (Figure 6c), both the mean and the standard deviation were significantly reduced, but noise near 25 cm distance remained present. This behavior was only observed for body-worn sensors, leading to the conclusion that these are motion artifacts caused by mechanical effects on the sensor element. Examples of this behavior are also visible in the presented time signals (Figure 5, A1-S1).

Consequently, the spatial calibration method is an effective way to improve distance estimation performance, as demonstrated by the results of 1.1 cm and 0.4 cm MAE. However, some limitations remain as the method was set up to work with a limited amount of training data. Since not all positions and orientations in space were equally represented during training, a change in device positioning may require additional validation to ensure similar performance. Additionally, the applied model with 27 multiplicative weights per actuator–sensor pair is still very rudimentary and small. While the approach is able to correct a misalignment in sensor element orientation (due to sensitive axis tilt or assembly tolerances), other expected disturbances, such as a positional deviation of the sensor elements, are not covered. Other performance-limiting factors, such as sensor/environment noise (range) and motion-induced noise, can only be addressed by hardware improvements or noise reduction algorithms [37].

Additionally, there are limitations to the applied validation method, even though OMC systems claim sub-millimeter accuracies in position estimation. Since we introduced some additional error by calculating the sensor center from the optical data, we believe that the best-performing magnetic estimates between 1 mm to 2 mm MAE are close to the optimum achievable in this experimental setup.

This study shows that the application of ME sensors for motion tracking works in principle and suggests characterization and calibration to overcome some of the limitations. However, ME sensors are not currently available as a market-ready product or in the quantities required for large-scale studies. As most of the signal processing aspects, including the spatial calibration, are sensor technology neutral, commercially available magnetometer alternatives could be used as an interim solution.

While this study was able to demonstrate the magnetic tracking during gait with sub-centimeter accuracy, it was not yet focused on extracting specific clinical gait parameters. Nevertheless, the time-varying distance between the lower extremities during walking is highly relevant for clinical movement analysis as it is related to step length and width. Deriving these parameters from distance estimates between both feet could be achieved with further research on targeted detection and analysis algorithms. Otherwise, state-of-the-art IMU-based estimation methods achieve centimeter-level accuracies based on dynamic time warping [8] or deep learning [46]. It is reasonable to assume that a direct assessment of distances between the lower extremities with the demonstrated accuracy leads to performance improvements if provided for a sensor fusion approach.

## 5. Conclusions

In this paper, we demonstrated a novel ME sensor-based magnetic motion tracking system and validated its performance against an optical reference. In a pilot study, five healthy subjects were equipped with the experimental setup comprised of body-worn and stationary magnetic devices. While tracking distances between the lower extremities, we achieved an accuracy of less than 1 cm under real-life-like conditions caused by treadmill walking at 1 m/s. These results were achieved with a comprehensive calibration scheme to overcome limitations of the early-stage ME sensor technology. This technical validation showed that the system is well suited for relative human motion tracking in terms of modularity, range, and accuracy. While the system is not as accurate as OMC systems (sub-millimeter), it is competitive with IMU-based approaches (few centimeters). Therefore, it is promising as a standalone gait analysis solution or in a sensor fusion approach with established wearable sensors. This should be further investigated with the goal of assessing a specific clinical gait parameter.

## Figures and Tables

**Table 1:** Validation of the magnetic distance estimation approach for Scenario A (arbitrary motion) and Scenario B (gait) in terms of the mean absolute error (MAE). Either no spatial calibration was applied (no training, sensor-specific enhancement only) or training data from one or both scenarios was used. Mean MAE refers to the MAE over all experiments and actuator–sensor pairs in this scenario. Max. MAE refers to the worst-performing actuator–sensor pair of all experiments in this scenario.

		Validation Datasets			
		Scenario A		Scenario B	
		Mean MAE	Max. MAE	Mean MAE	Max. MAE
**Training datasets**	Without training	1.4 cm	2.4 cm	1.0 cm	1.8 cm
	Scenario A	1.2 cm	1.9 cm	1.0 cm	2.9 cm
	Scenario B	1.5 cm	2.5 cm	0.4 cm	1.2 cm
	Scenarios A and B	1.1 cm	1.4 cm	0.4 cm	1.0 cm

## Data Availability

All motion data collected in this study are publicly accessible in the Zenodo repository ‘Magnetic Distance Estimation Data from Gait Experiments with Magnetoelectric Sensors’ [47]. The data are structured in the Motion-BIDS format for reproducible research [48,49].

## Abbreviations

The following abbreviations are used in this manuscript:

ME	Magnetoelectric
PD	Parkinson’s disease
OMC	Optical motion capture
IMU	Inertial measurement unit
LiDAR	Light detection and ranging
UWB	Ultra-wideband
NLOS	Non-line of sight
MEMS	Micro-electromechanical system
BIDS	Brain imaging data structure
OFDMA	Orthogonal frequency-division multiple access
PCB	Printed circuit board
LTI	Linear time-invariant
IIR	Infinite impulse response
MAE	Mean absolute error
(R)MSE	(Root) mean squared error
SNR	Signal-to-noise ratio

## Appendix A

**Table A1:** Equalizing filter parameters for each sensor element within a sensor node as estimated by the parameter sweep method described in Section 2.2.

Sensor Element [Internal ID]	Center frequency (Hz)	Bandwidth (Hz)	Gain (dB)	Sensitivity (kV/T)
S0 - X [D4]	7485.8	8.8	−40	131.9
S0 - Y [C1]	7514.3	9.2	−39	87.1
S0 - Z [B7]	7564.0	19	−41	108.1
S1 - X [G2]	7669.1	9.2	−44	192.8
S1 - Y [C5]	7666.0	10.4	−44	161.1
S1 - Z [G5]	7685.4	9.9	−45	98.6
S2 - X [C1]	7677.6	8.2	−53	38.7
S2 - Y [B1]	7681.1	10.1	−51	52.3
S2 - Z [A4]	7712.2	10.9	−51	15.7
S3 - X [C2]	7687.0	13.6	−48	20.7
S3 - Y [B1]	7708.3	8.5	−53	28.1
S3 - Z [B2]	7712.9	16.5	−48	20.5
